# *Conus regius*-Derived Conotoxins: Novel Therapeutic Opportunities from a Marine Organism

**DOI:** 10.3390/md20120773

**Published:** 2022-12-10

**Authors:** Francesco Margiotta, Laura Micheli, Clara Ciampi, Carla Ghelardini, J. Michael McIntosh, Lorenzo Di Cesare Mannelli

**Affiliations:** 1 Department of Neuroscience, Psychology, Drug Research and Child Health—NEUROFARBA, Pharmacology and Toxicology Section, University of Florence, 50139 Florence, Italy; 2 George E. Wohlen Veterans Affairs Medical Center, Salt Lake City, UT 84148, USA; 3 Department of Psychiatry, University of Utah, Salt Lake City, UT 84108, USA; 4 School of Biological Sciences University of Utah, Salt Lake City, UT 84112, USA

**Keywords:** alpha-conotoxins, nAChRs, alpha9alpha10 nAChRs, RgIA, RgIA analogues, RegIIA, RgIB, M-conotoxins, pain, neuropathic pain

## Abstract

*Conus regius* is a marine venomous mollusk of the *Conus* genus that captures its prey by injecting a rich cocktail of bioactive disulfide bond rich peptides called conotoxins. These peptides selectively target a broad range of ion channels, membrane receptors, transporters, and enzymes, making them valuable pharmacological tools and potential drug leads. *C. regius*-derived conotoxins are particularly attractive due to their marked potency and selectivity against specific nicotinic acetylcholine receptor subtypes, whose signalling is involved in pain, cognitive disorders, drug addiction, and cancer. However, the species-specific differences in sensitivity and the low stability and bioavailability of these conotoxins limit their clinical development as novel therapeutic agents for these disorders. Here, we give an overview of the main pharmacological features of the *C. regius*-derived conotoxins described so far, focusing on the molecular mechanisms underlying their potential therapeutic effects. Additionally, we describe adoptable chemical engineering solutions to improve their pharmacological properties for future potential clinical translation.

## 1. Introduction

Cone snail venom was first studied in the 1960s in order to understand the pharmacological basis for fatal envenomation resulting from cone snail stings [1,2,3]. These studies showed *Conus* venom to be a complex mixture of neuroactive peptides termed conotoxins. Since then, interest in these peptides has continued to grow for their possibilities both as natural drug leads for treatment of human diseases and as pharmacological probes for the characterization of several receptors. Today, the genus *Conus* comprises more than 750 species and each of them produces venom containing hundreds to thousands conotoxins [4]. In this review, we focused our attention on *Conus regius* venom, because it harbours a variety of conotoxins with powerful pharmacological properties. As each species of the *Conus* genus, *C. regius* exploits its venom to selectively capture prey, immobilizing it through molecules affecting the nervous system [5,6,7]. To date, the characterized peptides from *C. regius* consists essentially of α-conotoxins and mini-M conotoxins. While the activities of the latter are still unknown, α-conotoxins target nicotinic acetylcholine receptors (nAChRs), including α9α10, α3β2, α3β4, and α7 subtypes [8,9]. Since the signalling of these receptor subtypes is involved in various illnesses, *C. regius*-derived conotoxins are currently under investigation to better explore their potential uses, mainly in the treatment of pain, but also for cognitive disorders, drug addiction, and cancer. We report here the pharmacological characteristics of the most important *C. regius*-derived conotoxins described so far. Furthermore, we describe the strategies used to improve their activity, selectivity and stability for future, possible, therapeutic applications.

## 2. *Conus regius* and Its Venom Composition

*C. regius*, or the royal cone, is a venomous mollusk that harpoons its prey. These cone snails specialize on amphinomid polychaetes, or “fireworms”, that have painful stinging bristles, as their primary prey. *C. regius* is one of the most widespread and common species of the Caribbean region. These predators live in shallow to moderately deep waters, and are found in Florida and south through the Antilles and the Central American coast to central Brazil. *C. regius* is part of a subgenus of species known as *Stephanoconus*; these cones have strong tubercles on the spire of their shell leading to being referred to as regal in appearance [10].

Venom composition of *C. regius* was analyzed for the first time by Vianna Braga et al., in order to investigate the correlation between feeding behavior and intra-specific differences in venom. High performance liquid chromatography (HPLC) did not reveal a significant pattern of variation in the venom composition of different samples. Interestingly, although 104 peaks were detected, only one was selected for characterization based on its homogeneity throughout the different populations, its estimated solubility, its relative abundance in the profile, and its molecular weight. *De novo* sequencing showed a novel peptide belonging to the I- gene superfamily of conotoxins and having the XI framework (C-C-CC-CC-C-C), named rg11a [7]. Unfortunately, there have been no further studies about its biological activities. Thus, by describing the first conotoxin from *C. regius,* the authors have laid the groundwork for starting to study the biologically active toxins derived from this marine species.

Subsequently, other new conotoxins (reg1a–f, RgIA, RegIIA, RgIB and mini-M conotoxins) from *C. regius* were discovered using HPLC fractionation, PCR-based methods, and RNA sequencing [11,12,13,14,15]. As discussed below, some of these newly discovered conotoxins were used in their natural form or chemically synthesized. In addition, several synthetic derivatives were generated in order to improve the pharmacological characteristics of the peptides.

## 3. Conotoxins

Conotoxins are small disulfide-rich peptides that have different targets, including ion channels, G protein-coupled receptors (GPCRs), transporters, and enzymes [16,17]. Generally, conotoxins can be divided into several subfamilies, depending on their signal peptide sequence, cysteine framework pattern, and biological target [18]. The comparison of signal peptide sequences allowed definition of 30 groups, the gene superfamilies, that share higher sequence similarity. These groups are represented by Latin letters, such as A, D, I, J, M, O, P, S, T, and others [19]. Cysteine residues, which stabilize the secondary structure of such peptides [20], are highly conserved and useful to categorize conotoxins into 32 cysteine frameworks families, represented by Roman numbers (I–XXX, XXXII, and XXXIII) (Table 1) [4,19,21].

Furthermore, Greek letters are used to divide conotoxins in 12 pharmacological families (α, γ, δ, ε, ι, κ, μ, ρ, σ, τ, χ, ω), according to their targets [9,21,22,23]. However, the classification system is well described and analyzed in ConoServer (Institute of Molecular Bioscience IMB, Brisbane, Australia), a database specializing in the sequence and structures of conopeptides (http://www.conoserver.org/index.php, accessed on 2 November 2022) [19,22,24].

## 4. α-Conotoxins and Their Targets

α-Conotoxins are the largest group of characterized conotoxins, due to their abundance and pharmacological profile. Normally, α-conotoxins act as antagonists of neuronal and/or muscular nAChRs [8,9]. nAChRs are pentameric ligand-gated ion channels composed of α (α1–α10) and non-α (β1–β4, γ, δ, and ε) subunits. Each receptor is composed of five individual subunits, giving rise to an extensive range of combinatorial arrangements [25]. The α1, β1, δ, ε, and γ subunits form the nAChR subtypes (adult and fetal) found at the neuromuscular junction. The remaining α and β subunits assemble in various combinations to form numerous and distinct receptor subtypes, mainly, but not exclusively, expressed by nervous system cells [26]. nAChRs are actively being investigated as targets for pain and inflammation treatments [26].

α-Conotoxins activities for nAChRs are based on constituent amino acid residues and, particularly, intramolecular interaction among the cysteines. The most common cysteine framework of α-conotoxins is framework I, with four cysteines (C) arranged in the C_1_C_2_-X_n_-C_3_-Y_m_-C_4_ pattern, where X_n_ is a loop of amino acids with *n*= 3–4 and Y_m_ is a loop of amino acids with *m* = 3–8 [4]. The number and nature of the amino acids in these loops classify α-conotoxins in 3/5, 4/3, 4/4, 4/6, 4/7, and 4/8 subtypes, each of which has a different binding and selectivity towards the nAChRs subtypes [8,27]. Furthermore, depending on the disulfide bond connectivity, three possible isomers can be formed, namely globular (C_1_-C_3_, C_2_-C_4_), ribbon (C_1_-C_4_, C_2_-C_3_) and bead (C_1_-C_2_, C_3_-C_4_) [4,9]. Other less common cysteine patterns of α-conotoxins are the II, III, IV, VII, XIV, XX, and XXIV frameworks (Table 1) [4].

**Figure 1:**
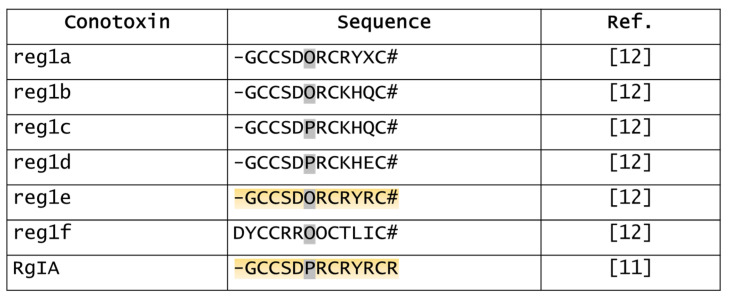
Sequences of reg1a–f and RgIA. X, unidentified amino acid; O, hydroxyproline; #, amidated C-terminal. Grey, note that Pro6 is hydroxylated in reg1a,b,e,f; orange, note the high sequence homology between reg1e and RgIA [11,12].

Reg1a–f were the first α4/3-conotoxins isolated from *C. regius* by HPLC (Figure 1) [12]. Sequences of reg1a,b,e,f are unusual as they are post-translationally hydroxylated on proline residues to produce hydroxyprolines (Hyp or O): reg1f has two Hyp, whereas reg1a,b,e have only one Hyp. Reg1b,c have the same sequence, but reg1c is not hydroxylated at Pro6. Reg1c,d only differ in residue 11 (Gln in reg1c vs. Glu in reg1d) [12]. Overall, reg1a–e have significant sequence homology, while reg1f has no homology. Hydroxylation enhances the polarity and hydrogen-bonding capabilities to these conotoxins and probably defines their mode of binding to nAChRs [12]. However, no biological activities of these conotoxins have been described, except for reg1e. Reg1e (50 nM) caused a 40% inhibition of acetylcholine-evoked currents in hybrid human (h) α9/rat (r) α10 (hα9/rα10) nAChRs [28]. Moreover, it inhibited voltage-gated calcium channel (VGCC) in rat dorsal root ganglia (DRG) neurons via γ-aminobutyric acid type B receptor (GABA_B_R) activation (IC_50_ 20.5 nM) [28]. Interestingly, RgIA, which has similar biological activities (see below), is supposed to be the precursor sequence of reg1e [11,28,29].

## 5. RgIA

**Figure 2:**
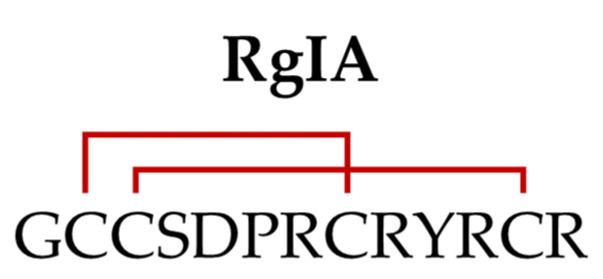
Sequence of RgIA (with indication of the two disulfide bonds).

α-RgIA (or RgIA) is the most documented α-conotoxin described in *C. regius.* RgIA was identified using a PCR-based discovery method, which later allowed its synthesis using standard Fmoc chemistry [11]. This peptide is characterized by the cysteine framework I (CC-C-C) and displays a globular disulfide connectivity (Figure 2) [30].

More specifically, the four amino acids in the first loop and the three in the second loop place it in the α4/3 subfamily of conotoxins [11]. RgIA was first tested as an antagonist on *Xenopus* oocyte expressing rα9α10, rα7, rα2β2, rα2β4, rα3β2, rα3β4, rα4β2, rα4β4, and rα6β2β3 nAChRs, showing a 1000–2000-fold greater potency at inhibiting the rα9α10 subtype compared to the others. Then, its high specificity for rα9α10 nAChR was also confirmed on the native channel expressed by inner hair cells [11]. The high affinity block of rα9α10 nAChRs by RgIA has been attributed, in part, to residues Asp5, Pro6; Arg7 in loop I and Arg9 in loop II [31].

The α9α10 nAChR subtype, which is critical for mediating synaptic transmission from the medial olivocochlear to the cochlear hair cells, has also been implicated in a series of pathological conditions, including neuropathic pain [26,32,33,34,35,36], tumor proliferation [37,38,39], and immune-mediated disorders [40,41,42,43,44]. Considering the high affinity of RgIA towards this receptor subtype [11,45], its discovery led to the possibility of studying the role of α9α10 nAChRs in several diseases.

### 5.1. Analgesic and Disease-Modifying Effects of RgIA

RgIA is reported as a potent pain-relieving compound in rat models of neuropathy. A single intramuscular injection of RgIA (0.02 and 0.2 nmol) produced an acute, dose-dependent, antihyperalgesic effect in a chronic constriction injury (CCI) rat model. Repeated daily injections of 0.2 nmol of RgIA, for 5 days, resulted in a sustained analgesic effect and decreased inflammation at the sciatic nerve [46]. Daily administration of RgIA (2 and 10 nmol) for 7 and 14 days provided acute antinociceptive effects on both days in CCI rats, with long-lasting effects evident only by day 14 [33]. Additionally, treatment with RgIA attenuated the degree of inflammation in the sciatic nerve and DRG, reducing edema and infiltrate. Both dosages of RgIA also significantly decreased the density of glial cells in the spinal cord and prevented morphological derangements in DRG [33]. The same α-conotoxin doses also showed pain-relieving and neuroprotective properties in a rat model of oxaliplatin-induced neuropathy [35]. The repeated administration (2 and 10 nmol, intramuscularly) reduced the oxaliplatin-induced neuropathic pain hyperalgesia and allodynia, and DRG damage. In the spinal cord, the numerical increase of astrocyte cell density present in oxaliplatin-treated rats was partially prevented by RgIA treatment. Nevertheless, the administration of the α-conotoxin was able, per se, to elicit a numerical increase and a morphological activation of microglia and astrocytes in specific brain areas [35], suggesting that RgIA may modulate glial cells in order to promote neurorestoration and reduce pain. Supporting this evidence, the administration of RgIA (2 nmol) for two weeks in oxaliplatin-treated rats elicited similar antinociceptive effects, avoiding DRG morphological alterations [47] These observations were consistent with a progressive disease-modifying effects in neuropathic context by RgIA. The presence of α9α10 nAChRs in immune cells [42,48,49,50] has led to the hypothesis that the long-term effects on neuropathic pain produced by RgIA might be due to the modulation of immune cells infiltration and the consequent release of inflammatory mediators into the site of injury, which may sensitize nerves to nociceptive stimuli [51,52].

However, RgIA was also proposed to exert its analgesic effects by modulating the N-type VGCC Ca_V_2.2 [53,54,55,56]. This inhibition is mediated by the activation of GABA_B_R, and block also occurs in DRG neurons of α9 knockout (KO) mice [54], suggesting that the α9α10 subtype is not involved in the modulation of the calcium channels. Inhibition of N-type channels, in turn, decreases neurotransmitter release and synaptic transmission between DRG neurons and second-order neurons in the spinal cord and thus impairs the transmission of nociceptive signals to the brain [57]. This is the mechanism of action of another well-known *Conus*-derived compound, ω-conotoxin MVIIA, an FDA approved drug known as Prialt (Elan Pharmaceuticals, Dublin, Ireland), for the treatment of chronic pain [58,59].

RgIA (0.1 μM) was shown to inhibit VGCC currents by 40–50% in >75% of DRG neurons isolated from either mice or rats [54,55], an effect that was prevented by competitive GABA_B_R antagonists [55] or the knockdown of the GABA_B_R by siRNA [56]. Consequently, GABA_B_R seems to be necessary for the inhibition of currents by the conotoxin. RgIA (10 μM) does not displace the binding of the antagonist [3 H]-CGP54626 to human GABA_B_R transiently transfected in HEK293T [60], suggesting a non-competitive mechanism. The GABA_B_R also functionally couples to the G protein coupled inwardly rectifying potassium (GIRK) channels to attenuate nociceptive transmission [61]. Recently, it has been shown that RgIA (1 μM) potentiates inwardly rectifying potassium currents in HEK293T cells heterologously expressing human GABA_B_R coupled to GIRK1/2 channels [62], albeit it failed to elicit the same effects in *Xenopus* oocytes in a previous study [60]. In support of the latter evidence, Wright et al. [63] demonstrated that RgIA (0.1–1 μM) also had an insignificant effect on VGCC currents (inhibition >10% in <20% of rat DRG neurons), showing contrasting data to those obtained by Callaghan et al. [55]. Moreover, no correlation was found between the responses induced by RgIA and baclofen, a GABA_B_R agonist. By activating the presynaptic GABA_B_R in DRG neurons, baclofen inhibits excitatory post-synaptic currents (EPSCs) and prevents the release of glutamate, which blocks pain transmission between the primary nociceptors and second-order neurons in the dorsal horn of the spinal cord [34,64]. α-Conotoxins are charged peptides, so they may not cross the blood–brain barrier and reach spinal neuron synapses, suggesting that the VGCC mechanisms are not involved in RgIA-induced analgesia [34,60,63]. These findings are consistent with a study that demonstrated no inhibition of EPSCs in the dorsal horn neurons by Vc1.1, another peptide of the α-conotoxin family [64].

There are several conflicting results that make it difficult to determine the molecular mechanism by which α-conotoxins exert their analgesic effect. Many studies suggested that RgIA and some other α-conotoxins do not relieve pain by the activation of GABA_B_R, reinforcing the idea of the involvement of α9α10 nAChRs [34,36,60,63,64]. As mentioned above, RgIA and other α9α10 nAChRs antagonists have been associated with disease-modifying effects in neuropathic pain conditions [9,33,34,35,36,60]. Known GABA_B_R agonists, such as baclofen, did not show similar properties. RgIA4 (see below for its amino acid composition), a RgIA derivative that lacks GABA_B_R activity, maintains the capacity to prevent the development of neuropathic pain [36,65,66]. Moreover, RgIA, which induces calcium transients in murine granulocytes, decreased reactive oxygen species production [42,44] and increased the production of the anti-inflammatory interleukin-10 in these cells. Even in this case, the α9α10 nAChR mechanism is more suitable than the GABA_B_R mechanism for explaining such regulation, given that the inhibition of VGCC currents mediated by GABA_B_R might result in lowering intracellular calcium, not in its increase [42]. Finally, evidence from α9 subunit KO mice also supports a role for α9α10 nAChRs in pain [36,67,68].

### 5.2. Anti-Colitis Effects of RgIA

In addition to analgesic and disease-modifying activities in animal models of pain, AlSharari et al. reported for the first time the anti-inflammatory effects of RgIA in the dextran sodium sulfate (DSS) experimental mice colitis model. Briefly, the lower doses (0.02 and 0.1 nmol) of the RgIA treatment in DSS-treated mice were inactive, whereas the higher dose (0.2 nmol) reversed the disease activity index score, loss of body weight, total histological damage score, as well as the colonic increase of the TNF-α concentration compared to the control group. Moreover, 0.2 nmol of RgIA significantly prevented the colon length shortening in DSS-treated mice [40]. The block of α9α10 nAChRs on gut immune cells as an anti-inflammatory signal was suggested as a pharmacodynamic mechanism.

### 5.3. Anticancer Effects of RgIA

As mentioned above, nAChRs also have a major role in tumorigenesis and cancer progression [37,38,39]. The expression of α9 and/or α10 subunits has been reported in several cancer cell lines, human tumors, and immune cells that could promote cancer-related inflammation [42,48,49,50,69,70,71,72]. On these bases, α9α10 nAChR blockers were studied as antitumor agents. Intraperitoneal injections of 0.1 nmol/kg of RgIA led to changes of a degenerative nature of both cancer cells and leukocytes infiltrating the tumor in Ehrlich carcinoma (EC)-bearing mice [43]. Interestingly, the α-conotoxin (1 nmol/kg) also enhanced the antitumor activity of splenocytes and increased the survival rate of EC-bearing mice by impairing tumor growth [73]. In vitro, RgIA increased the cytotoxic effects of the lipoxygenase pathway inhibitors nordihydroguaiaretic acid (24 h of treatment) and baicalein (48 h of treatment) on EC cells, which display an increased activity of the arachidonic acid cascade [74]. By contrast, a recent report showed that while baicalein exerted a significant antiproliferative and cytotoxic activities against C6 glioma cells, RgIA enhanced the proliferation of these cells [75]. No additional studies on the use of RgIA as an anticancer agent are reported. RgIA may have contrasting effects on cell proliferation, depending on the concerned cell line. Better anticancer activity was reported for synthetic derivatives of RgIA, as described below for RgIA4 [71].

### 5.4. Derivatives of RgIA

**Figure 3:**
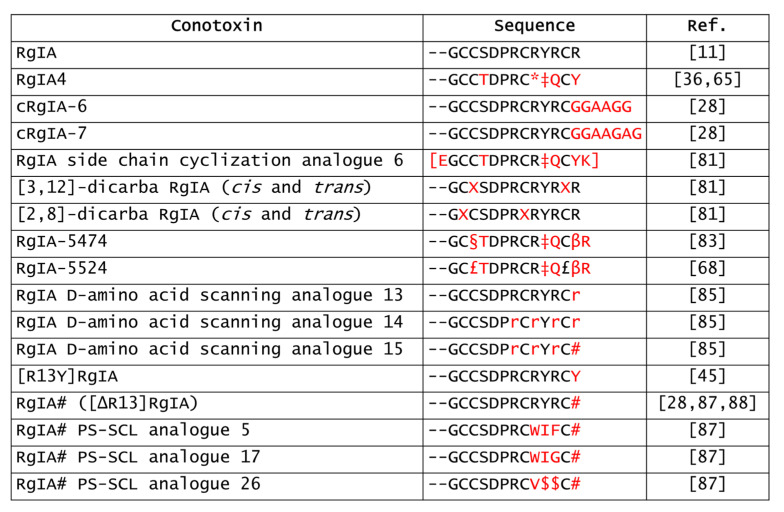
Sequences of the RgIA analogues reported in this review. *, citrulline; ‡, 3-Iodo-Tyrosine; [], side chain cyclization; X, positional sequence insertion of L-allylglycine residues prior to ring-closing olefin metathesis cyclization; §, L-penicilammine; β, 3-homo tyrosine; £, methylene thioacetal replacement; #, amidated C-terminal; r, D-arginine; $, norleucine [11,28,36,45,65,68,81,83,85,87,88].

Owing to its high specificity towards α9α10 nAChRs, RgIA also provided a promising lead for developing novel ligands that selectively target these receptor subtypes. Unfortunately, several α-conotoxins, including RgIA, were initially tested in rodent nAChRs-expressing systems or native receptors as well as preclinical pain studies were most often carried out on rodent models. The crystal structure of hα9 nAChR extracellular domain with RgIA revealed that RgIA also interacted with hα9α10 nAChRs, involving its Asp5-Pro6-Arg7 triad of loop I and Arg11 of loop II [76]. However, there is a 300-fold difference (IC_50_ rat 1.5 nM vs. IC_50_ human 490 nM) of RgIA potency between the rat and human receptors, due to the residue at position 56 (Ile in humans vs. Thr in rats) of the α9 subunit [77,78]. These species differences likely led to the failure of the clinical development of a potential hα9α10 antagonist based on Vc1.1 [79]. Hence, the derivatives of native α-conotoxins are needed to improve specificity and potency against hα9-containing nAChRs together with pharmacokinetics features (Figure 3).

RgIA4 is the most reported RgIA-analogue in the literature data. This peptide has 5 of the 13 total amino acids modified but shows an increased potency and selectivity towards the hα9α10 subtype, retaining a high affinity for both rat and human receptors (IC_50_ rat 0.9 nM vs. IC_50_ human 1.5 nM) [36]. With a 1200-fold lesser potency than that for α9α10, RgIA4 inhibits the hα7 subtype (IC_50_ 1.8 μM). Interestingly, RgIA4 lacks activity on GABA_B_R, allowing the dissection of the involvement of α9α10 vs. GABA_B_R in pain relief [36]. Daily subcutaneous injections of RgIA4 (0.128, 16 and 80 μg/kg) prevented cold allodynia and mechanical hypersensitivity in oxaliplatin-treated rats, after only one week of treatment [36]. The same treatment with 40 μg/kg of RgIA4 reversed oxaliplatin-induced cold allodynia in mice but only after 3 weeks of treatment. Interestingly, the effects lasted up to 3 weeks post-treatment, indicating a disease-modifying effect like that of the native peptide [65]. Long-lasting effects have also been reported in a paclitaxel-induced neuropathy rat model, where animals subcutaneously injected with RgIA4 (80 μg/kg) showed a significant reduction of mechanical allodynia by day 12 post-treatment [66]. These data support the effects of RgIA4 in multiple chemotherapy-induced neuropathy models. As anticipated above, RgIA4 (1 μM) completely abolished the proliferation of A549 adenocarcinoma cell line induced by 48 h treatment with 100 nM nicotine, blocking the nicotine-induced activation of Akt and ERK [71].

However, RgIA4 is not an ideal drug candidate because of its high in vivo degradation and poor serum stability [21]. Synthetic cyclization, with appropriately sized linkers between the N- and the C-termini, could be useful to overcome this issue. In fact, in a previous study, the insertion of a linker of 6 or 7 amino acids in the native RgIA (cRgIA-6 and cRgIA-7) caused an improvement in stability compared to the linear peptide, while maintaining a high potency towards the α9α10 nAChR [28]. By contrast, incorporating smaller linkers does not make significant improvements, suggesting that they are likely to force the N- and the C-termini together, putting a strain on the peptide conformation [28]. Similarly, the side chain cyclization of RgIA4 (analogue 6) led to an increased serum stability over linear RgIA4, while exhibiting a similar affinity with RgIA4 at the hα9α10 nAChR and analgesic effects in oxaliplatin-treated rats [80]. Structurally, the lactam linkage introduced in RgIA4 stabilized the globular conformation and suppressed disulfide scrambling, leading to stability improvements.

Disulfide bond replacement with a dicarba unsaturated bridge is a further strategy to enhance the serum stability, as well as to modulate the specificity of conotoxins [81,82]. Additionally, [3,12]-dicarba RgIA analogues retain inhibition at the α9α10 nAChR but lack GABA_B_R activity, whereas [2,8]-dicarba analogues display reverse target selectivity, suggesting that the C_1_-C_3_ bridge is important for inhibiting the α9α10 nAChR and the C_2_-C_4_ bridge for GABA_B_R modulation [81]. Molecular dynamics simulations suggested that substitution at Cys2 and Cys8 abolishes the RgIA activity at α9α10, mainly by the reduction of contacts between RgIA-Tyr10 and α9-Arg138 and between RgIA-Arg9 and α10-Trp81 [81]. The importance of the C_1_-C_3_ bridge on the activity of RgIA has also been demonstrated by two recent works. The substitution of the Cys3 with the Cys surrogate L-penicillamine, together with amino acids replacements and addition of L-Arg in position 14, resulted in RgIA-5474, a peptide with a 9000-fold increased potency towards the hα9α10 nAChR (IC_50_ 0.05 nM) and improved serum stability compared to RgIA [83]. To date, RgIA-5474 is one of the most potent RgIA-derivatives against hα9α10 nAChR. A fluorescently tagged derivative of this peptide has been synthesized using click chemistry and can be used to visualize native α9α10 nAChRs [84]. Furthermore, the replacement of the C_2_-C_4_ bridge with unreducible methylene thioacetal and the same above-mentioned amino acids modifications led to the generation of RgIA-5524, a further compound with highly selectivity for hα9α10 nAChR (IC_50_ 0.9 nM), reduced serum degradation and pain-relieving effects in oxaliplatin-treated mice (40 μg/kg) [68]. Hence, disulfide loop modifications could be helpful to improve biophysical properties of RgIA, by stabilizing its globular structure and avoiding disulfide scrambling.

In addition to the disulfide scrambling that makes its structure unstable, RgIA is more susceptible to proteolysis than other conotoxins due to its arginine-rich loop II and Tyr10, which provide cleavage sites for proteases. Ren et al. provided the solution by generating three RgIA D-amino acid scanning analogues (called peptides 13, 14, and 15) that are more resistant in serum and intestinal fluid than native RgIA [85]. D-amino acids are less prevalent in nature, thus they are unlikely to be susceptible to enzymatic degradation. Notably, peptide 15 displayed a two-fold increase in the inhibition of hα9α10 nAChR, whereas peptide 13 had a two-fold more potency against rα9α10 nAChR, compared to RgIA [85]. Peptide 13 retained its strong affinity because it contained D-enantiomer of Arg at the C-terminal, which is not involved in binding to α9α10 [31,85]. All the other synthesized D-enantiomer analogues showed decreased potency at the α9α10 receptor, indicating that amino acid chirality is important for the activity of these α-conotoxins. Separately, it was recently found that the substitution of Arg13 with Tyr ([R13Y]RgIA) significantly improved the potency of RgIA for hα9α10 nAChRs by 240-fold, nullifying the difference between rats and humans [45]. Thus, scan strategies are useful in revealing the critical amino acid involved in the peptide-receptor interaction. Positional-scanning synthetic combinatorial libraries (PS-SCL) consist of a mixture of peptides where one or more of the positions are individually fixed at a specific amino acid, while the remaining positions are comprised of an equimolar mixture of amino acids [86]. PS-SCL of RgIA# ([∆R13]RgIA) based on positions 9, 10, and 11 provided 10,648 possible conotoxin sequences, but, in the end, only three lead compounds (5, 17, and 26) displayed increased antinociception compared to the native peptide when intraperitoneally injected in mice (10 mg/kg) [87].

Finally, a further way to enhance the activity of α-conotoxins is dimerization. PEG9-dimeric RgIA# resulted in increased inhibitions at hα7 (IC_50_ dimeric RgIA# 63.1 nM vs. IC_50_ RgIA# >1000 nM) and hα9α10 (IC_50_ dimeric RgIA# 38.5 nM vs. IC_50_ RgIA# 248.7 nM), by 17- and 7-fold, respectively, compared to RgIA# [88]. Considering the role of these nAChRs in the development of cancer [71], dimeric RgIA# could be a useful pharmacological tool in cancer research. The PEG-linker also allows the conotoxin to simultaneously bind two adjacent binding sites, and further elongation of the linker should not significantly affect the activity of the dimer. Indeed, the PEG6-dimeric RgIA# showed comparable potency to the PEG9-dimeric RgIA# [88].

Overall, chemical modifications of α-conotoxins may not only be helpful to improve their pharmacokinetic and pharmacodynamic features, but also to elucidate the molecular mechanisms underlying their activities, in order to facilitate their use as drug leads.

## 6. RegIIA

**Figure 4:**
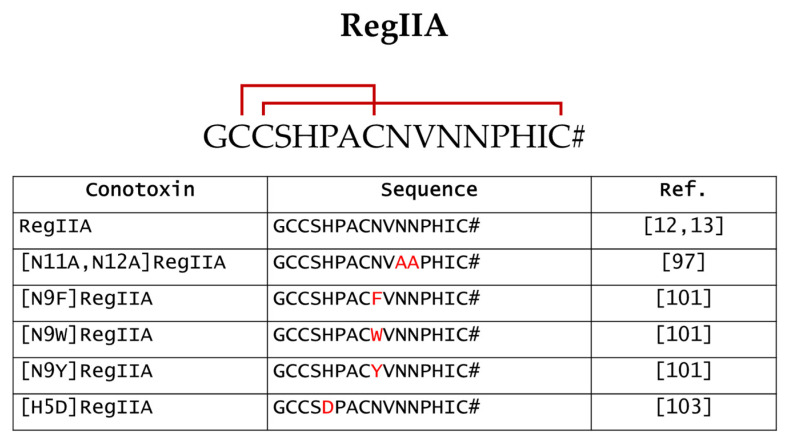
Sequences of RegIIA (with indication of the two disulfide bonds) and the RegIIA analogues reported in this review. #, amidated C-terminal [12,13,97,101,103].

RegIIA was isolated from *C. regius* venom using a HPLC-based strategy and its amino acid sequence was obtained by Edman degradation [12,13]. Limited amounts of the natural peptide precluded further investigations, thus RegIIA was later chemically synthesized [13]. It belongs to the α4/7 subclass of α-conotoxins having the conserved cysteine framework I (CC-C-C). Due to its C_1_-C_3_ and C_2_-C_4_ disulfide connectivity (Figure 4), RegIIA exhibits a classical globular structure [13].

The signal sequence of RegIIA places it in the A-superfamily of conotoxins. RegIIA potently inhibited acetylcholine-evoked currents of rα3β2 and rα3β4, and hα7 (IC_50_ values of 33, 97, and 103 nM, respectively), whereas it did not inhibit rα4β2, rα9α10, and muscle nAChRs (IC_50_ > 1000 nM) [13]. For these reasons, despite the poor selectivity, RegIIA is considered one of the most potent antagonists of α3β4 nAChRs.

Neuronal nAChRs play an important role in the central and peripheral nervous systems, where they are functionally involved in several physiological and pathophysiological processes [89]. For example, the α3β4 nAChR has been shown to be involved in lung cancer, drug abuse, and nicotine addiction, whereas the α3β2 subtype is related to disorders associated with specific areas, such as the medial habenula, cerebellum, spinal cord, retina, and autonomic ganglia [90,91,92,93]. However, α3β4 and α3β2 subtypes have also been detected in non-neuronal cells, such as human vascular endothelial cells, epithelial cells, immune cells, keratinocytes, fibroblasts, and glial cells [94,95,96], where they modulate a number of biological processes, such as cell proliferation, migration, differentiation, survival, and gene expression [95,96]. Therefore, α3β4 and α3β2 subtypes could be promising therapeutic targets, making attractive pharmacologically active substances modulating these nAChR subtypes.

To date, however, the effect of RegIIA on nAChRs-related diseases has not yet been evaluated in any preclinical studies, suggesting that improvements in selectivity are needed to make it a potential lead compound. Below, we reported some of the chemical modifications to enhance the selectivity and sensitivity of RegIIA (Figure 4).

### 6.1. Strategies to Improve RegIIA Selectivity towards Specific nAChRs Subtypes

Although native RegIIA is a potent inhibitor of the α3β4, it cannot be considered a selective pharmacological probe. Alanine scan mutagenesis approach revealed the double alanine-analogue [N11A,N12A]RegIIA inhibited the rα3β4 nAChR subtype with a seven-fold less potency than native RegIIA but, at the rα3β2 and hα7 nAChR subtypes, the potency decreased by 1000- and 360-fold, respectively, thus indicating an increase in the selectivity for the rα3β4 subtype [97]. Concerning human nAChR subtypes, RegIIA exhibited a higher selectivity towards hα3β4 than hα3β2 [98,99]. Receptor mutagenesis sustained the importance of loop D residue 59 and loop E residue 113 in nAChRs as key residues for RegIIA affinity. Specifically, two mutations, [T59K]α3β2 and [S113R]α3β2, strongly enhanced the α-conotoxin affinity compared with wild-type α3β2, whereas opposite point mutations in α3β4 exerted the contrary effect [98]. Moreover, a recent study showed that oligosaccharide chains on hα3β4 nAChR are crucial for interaction with RegIIA and that His14 plays an important role for the structure-activity relationship of this conotoxin [100].

Since specific RegIIA analogues for α3β2 have not yet been developed, a computational-based scanning approach has shown that introducing an aromatic residue to position 9 of RegIIA ([N9F], [N9W], and [N9Y]RegIIA) led to an increased potency against hα3β2 compared to hα3β4, identifying in [N9Y]RegIIA the most selective inhibitor [101]. In this way, it would be possible to study the role of the α3β2 subtype in nAChR-related diseases.

### 6.2. Species-Specific Differences in RegIIA Sensitivity

As mentioned above, the characterization of α-conotoxins in heterologous expression systems using mainly rat-cloned nAChR subunits is an obstacle to the effective development of nAChR subtype-selective α-conotoxins as pharmacological tools with therapeutic potential. In fact, conotoxins are likely to exhibit a different potency profile against the nAChRs subtypes of different species.

RegIIA, characterized as a competitive antagonist of rα3β2 and rα3β4 nAChRs, is 70-fold less potent at the homologous hα3β2 subtype [99]. This change was even more prominent for [N11A,N12A]RegIIA, which was inactive at the hα3β2 subtype but retained micromolar potency at rα3β2 (IC_50_ 9.9 µM) [97]. At α3β4, neither RegIIA nor the analogue, showed species-specific differences in sensitivity, suggesting that the determinants for the species difference would reside in the β2 subunit [97,99]. Surprisingly, when the non-homologous residues in the rat β2 subunit were exchanged to the human counterpart, a non-significant loss in sensitivity to RegIIA was observed. Receptor mutagenesis and molecular dynamics studies indicated that this difference can be mainly attributed to a single amino acid residue in position 198 on the rat α3 nAChR subunit. Consequently, a single exchange of residue Gln198 in the rat α3 subunit led to a loss of potency consistent with those of hα3β2 nAChRs. This residue exchange causes structural changes at the agonist binding site, reducing the interaction with RegIIA residues [99].

Another species-specific difference concerns the α7 nAChRs, which play an important role in cancer development [71] and in cognitive disorders, such as Alzheimer’s disease and schizophrenia [102]. As mentioned above, RegIIA is also an antagonist of the hα7 subtype [13], although it inhibits the rα7 subtype five-fold more powerfully [103]. The aspartate analogue [H5D]RegIIA strongly increased to 130-fold the difference in this species selectivity, which has been shown to be influenced by the residue in position 185 on the receptor [103].

Hence, it can be understood that mutagenesis studies coupled with molecular dynamics simulations can lead to the customized design of conotoxin analogues, which may be useful in increasing their specificity not only towards a specific nAChR subtype of the receptor, but also towards a species-specific subtype.

## 7. RgIB

**Figure 5:**
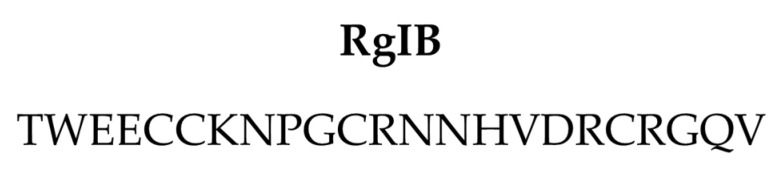
Sequence of RgIB.

α-RgIB (or RgIB) is another 4/7 α-conotoxin characterized by cysteine framework I (CC-C-C) (Figure 5) [14]. This conotoxin was isolated from the *C. regius* venom through HPLC fractionation and purification. To date, neither the features of its disulfide connectivity nor to which gene superfamily it belongs are known. The unique report in the literature data showed two biological activities by RgIB. First, intracranial injections of RgIB (1 nmol) aroused hyperactive behavior in mice, while lower doses (0.1 and 0.5 nmol) caused breathing difficulty. Second, RgIB (10 μM) induced an irreversible blockage of carbamylcholine-induced currents by 40% in nAChRs-expressing cells (PC12 cells), acting very likely on α3β4 and α3β4α5 subtypes [14].

Without any other evidence about this α-conotoxin, it is currently not possible to establish with certainty its specific targets and related pharmacological effects. Further studies are needed to better characterize the pharmacological and structural properties of RgIB.

## 8. Reg3b and Mini-M Conotoxins

**Figure 6:**
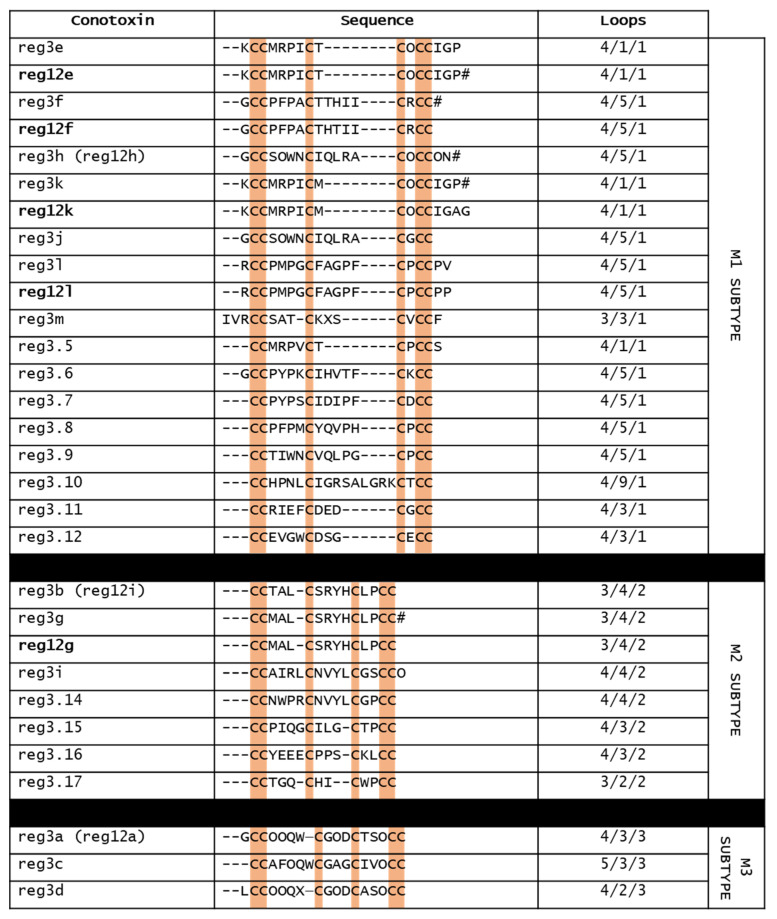
Sequences of all mini-M conotoxins from *C. regius.* O, hydroxyproline; #, amidated C-terminal; X, unidentified amino acid.

Conotoxins of the M-superfamily have been found in the venom of all *Conus* species [104]. Normally, M-superfamily conotoxins display a cysteine framework III (CC−C−C−CC), with a wide variability in the cysteine pattern and size of the three loop regions. Furthermore, compared to framework I of α-conotoxins, M-conotoxins having the framework III are constrained by three disulfide bonds. Within this gene superfamily, there are five M-subtypes (M1–M5), based on the number of amino acids in the third loop. M1–M3 conotoxins are grouped in “mini-M” (<22 residues), whereas M4 and M5 belong to the “maxi-M” group (>22 residues) [104,105]. While the latter are the better-known M-superfamily conotoxins, unfortunately little is known about mini-M conotoxins. Figure 6 shows the sequences of the mini-M conotoxins found in the venom of *C. regius*.

Reg12a,e–i,k,l were the first mini-M conotoxins isolated by HPLC and sequenced from the venom of *C. regius* [12]. These conotoxins did not have sequence homology and did not share the same post-translational modification pattern. Concerning their loop size, they were characterized by four variants (3/4/2, 4/1/1, 4/3/3, and 4/5/1), suggesting that their targets might be diverse. Reg12e,f,h,k,l belonged to M1 subtype, reg 12g,i to the M2 subtype, and reg12a to the M3 subtype [12]. More recently, further mini-M conotoxins have been found in *C. regius* venom duct by transcriptomic (reg3.5–12 and reg3.14–17) and peptidomic analysis (reg3a–m) [15]. Of these, only reg3a–m have been isolated by HPLC, because they are expressed in larger quantities.

These conotoxins exhibited variability in loop sizes, post-translational modifications, disulfide connectivities and three-dimensional folds, which are determinant for the final shape of the peptidic scaffolds and their activities. Based on loop sizes, seven of the isolated reg3 conotoxins belonged to the M1 group, three to the M2 group, and three to the M3 group [15]. Three-dimensional structure analysis was performed only on reg3b, the most abundant mini-M conotoxins of *C. regius.* Reg3b has 3/4/2 loop sizes and form three disulfide bonds between the first and sixth, second and fourth, and third and fifth cysteines, conferring a highly compact overall fold [15]. Aligning the sequences of mini-M conotoxins of *C. regius*, it was found that reg12a,h,i corresponded, respectively, to reg3a,h,b, whereas reg12e,f,g,k,l had high sequence homology, respectively, with reg3e,f,g,k,l. As already mentioned, neither the pharmacological activities of reg3b nor of the *C. regius*-derived mini-M conotoxins are known. However, given that ArchIIIA, a mini-M conotoxin from *C. archon* that differs from reg3b only in residue 3 (Ser in ArcIIA vs. Thr in reg3b), blocks hα7 nAChRs [106], it is possible that reg3b also shows this activity.

Overall, the plasticity of these mini-M scaffolds makes them interesting for the development of chemically modified peptides with therapeutic applications.

## 9. Conclusions

Given its amazing biodiversity and large number of yet-undiscovered species, the sea may be a rich source for new medicines. Nevertheless, only 20 drugs from marine organisms are currently in clinical use [107]. Conotoxins have attracted interest as great lead compounds for designing new drugs, due to their well-defined structure and ability to discriminate among subtypes of ion channels. In this review, we described the features of *C. regius*-derived conotoxins, which could represent promising therapeutic resources for the treatment of several diseases, including neuropathic pain, drug addiction, cognitive disorders, and cancer. Unfortunately, the development as drugs of many peptides is usually hindered because of their low stability and bioavailability. However, we also showed that the chemical engineering of conotoxins could be useful for improving their pharmacodynamic and pharmacokinetic properties, ensuring the concrete opportunity to develop new therapeutic agents starting from marine sources.

## Figures and Tables

**Table 1:** Cysteine Frameworks of Conotoxins.

Framework	Cysteine Pattern	Disulfide Bonds
I	CC-C-C	2
II	CCC-C-C-C	3
III	CC-C-C-CC	3
IV	CC-C-C-C-C	3
V	CC-CC	2
VI	C-C-CC-C-C	3
VII	C-C-CC-C-C	3
VIII	C-C-C-C-C-C-C-C-C-C	5
IX	C-C-C-C-C-C	3
X	CC-CXOC ^1^	2
XI	C-C-CC-CC-C-C	4
XII	C-C-C-C-CC-C-C	4
XIII	C-C-C-CC-C-C-C	4
XIV	C-C-C-C	4
XV	C-C-CC-C-C-C-C	4
XVI	C-C-CC	4
XVII	C-C-CC-C-CC-C	4
XVIII	C-C-CC-CC	3
XIX	C-C-C-CCC-C-C-C-C	5
XX	C-CC-C-CC-C-C-C-C	5
XXI	CC-C-C-C-CC-C-C-C	5
XXII	C-C-C-C-C-C-C-C	4
XXIII	C-C-C-CC-C	3
XXIV	C-CC-C	2
XXV	C-C-C-C-CC	3
XXVI	C-C-C-C-CC-CC	4
XXVII	C-C-C-CCC-C-C	4
XXVIII	C-C-C-CC-C-C-C-C-C	5
XXIX	CCC-C-CC-C-C	4
XXX	C-C-CCC-C-C-C-CC	5
XXXII	C-CC-C-C-C	3
XXXIII	C-C-C-C-C-C-C-C-C-C-C-C	N/D

^1^ X, any amino acid; O, hydroxyproline.

## Data Availability

Not applicable.
